# Low Preoperative Mean Arterial Pressure as a Risk Factor for Contrast-Induced Nephropathy After Rotational Atherectomy

**DOI:** 10.31083/RCM43418

**Published:** 2025-11-26

**Authors:** Xiaogang Liu, Lei Wan, Xinying Wu, Ye Gu, Liqun Hu

**Affiliations:** ^1^Department of Cardiology, Wuhan Fourth Hospital, 430033 Wuhan, Hubei, China

**Keywords:** contrast-induced nephropathy, rotational atherectomy, mean arterial pressure, MACCE

## Abstract

**Background:**

This study aimed to identify risk factors for contrast-induced nephropathy (CIN) following rotational atherectomy (RA) in patients with severely calcified coronary lesions to facilitate the prevention of CIN.

**Methods:**

A retrospective analysis was performed on 111 patients who underwent RA in Wuhan Fourth Hospital from July 2021 to June 2023. The creatinine levels of the patients were detected within 48–72 hours after RA, and the patients were divided into a CIN (n = 16) and a non-CIN group (n = 95). Propensity score matching was applied with a caliper value set at 0.02, resulting in 13 matched patient pairs. The risk factors for CIN after RA in these patients were analyzed.

**Results:**

A total of 16 cases of CIN occurred among the 111 patients with coronary heart disease who underwent RA. Following propensity score matching, 13 patients were included in both the CIN and non-CIN groups. The rates of heart failure were significantly higher in the CIN group than those in the non-CIN group before RA (all *p* < 0.05). However, there was no significant difference in preoperative mean arterial pressure (MAP) between the two groups. Nonetheless, the rate of patients with preoperative MAP <80 mmHg was higher in the CIN group than in the non-CIN group (53.8% vs. 7.7%; *p* < 0.05). The coronary artery lesion characteristics and interventional treatment strategies were comparable between the two patient groups. Moreover, no statistically significant difference was observed in 1-year major adverse cardiovascular and cerebrovascular events (MACCEs) or secondary endpoint events between the two groups. Logistic regression analysis showed that among the risk factors for CIN after RA, preoperative MAP <80 mmHg (odds ratio (OR) = 17.865, 95% confidence interval (CI): 1.135–281.246) was a risk factor for CIN (*p* < 0.05).

**Conclusion:**

Patients with a preoperative MAP below 80 mmHg are at increased risk of CIN following RA. This cohort requires intensive monitoring to prevent CIN, ensuring prompt implementation of management strategies to avert CIN onset and mitigate the adverse effects of CIN post-RA treatment.

## 1. Introduction

With rising percutaneous coronary intervention (PCI) volumes, particularly among elderly patients, PCI is increasingly extended to more complex vessel diseases. The number of severely calcified coronary lesions during PCI has increased significantly. Severely calcified coronary lesions are a significant clinical challenge for PCI. Stent implantation failure and incomplete expansion—common consequences of severe coronary calcium—significantly diminish PCI success rates longitudinally. Rotational atherectomy (RA) constitutes a cornerstone strategy for managing these complex lesions [1, 2]. The use of RA to pretreat severely calcified coronary lesions can improve the efficiency of interventional surgery, reduce complications of interventional procedures, increase the success rate of PCI [3], and may also reduce long-term in-stent restenosis.

Contrast-induced nephropathy (CIN) is characterized by an abrupt decline in kidney function precipitated by intra-arterial or intravenous contrast injection [4]. It is currently the third leading cause of acute renal deterioration in hospitals, following reduced renal perfusion and acute renal failure [1]. While interventional procedures pose minimal renal risk in the general population, substantially elevated nephrotoxicity hazards exist in vulnerable subgroups, notably among cardiac surgery patients. Reported rates can vary considerably between centers [2, 3, 4, 5, 6]. CIN post-PCI is related to increased risk of mortality and dialysis, as well as recurrent myocardial infarction and target vessel reconstruction [6]. Given the current absence of proven therapies for CIN combined with the scarcity of preventive strategies validated by randomized controlled trials, developing effective renal protective protocols remains a critical unmet need in interventional medicine [2]. Therefore, risk stratification, prevention, and treatment of CIN are of clinical importance, and CIN remains a hot topic of concern to clinicians.

Established predictors of contrast-induced nephropathy encompass pre-existing renal dysfunction, diabetic comorbidity, advanced age, and impaired cardiac function [7], among which hemodynamic instability is also an important risk factor. In particular, perioperative hypotension and the use of intra-aortic balloon pumps could increase the risk of CIN, which may be related to low mean arterial pressure (MAP) leading to insufficient renal perfusion [8, 9]. Abnormal blood pressure (including hypertension and hypotension) may affect the risk of CIN through multiple mechanisms. On the one hand, long-term hypertension can lead to renal vascular sclerosis, microcirculatory disorders, and endothelial dysfunction, weakening the kidney’s ability to compensate for ischemia and toxins. Epidemiological data show that the incidence of CIN in hypertensive patients is 1.5–2 times higher than that in normal blood pressure patients, and poor blood pressure control (such as systolic blood pressure >160 mmHg) is associated with more severe renal damage. On the other hand, perioperative hypotension (such as systolic blood pressure <90 mmHg) may cause insufficient renal perfusion and aggravate medullary hypoxia and oxidative stress caused by contrast media. This “double-edged sword” effect makes blood pressure monitoring and management a key issue for the prevention of CIN: it is necessary to avoid vascular damage related to hypertension, and to maintain adequate perfusion pressure to protect renal function [10]. Patients undergoing PCI for moderately or severely calcified lesions have an increased prevalence of renal insufficiency [11]. Caution in the usage of contrast media is appropriate in these cases to reduce the risk of CIN. Compared with ordinary PCI patients, patients undergoing RA often have multiple comorbidities, longer procedure time, and higher contrast media dose. At present, there are no relevant clinical reports on the impact of blood pressure on CIN after PCI in patients undergoing RA. This study retrospectively analyzed the risk factors for CIN in patients with coronary heart disease who underwent RA, focusing on the blood pressure indicators, providing related information that adds to the early screening, identification, and management decision-making of CIN in patients undergoing RA.

## 2. Materials and Methods

### 2.1 Study Subjects

This study was a single-center, retrospective study. A total of 111 patients with severe coronary artery calcification who underwent RA in the Department of Cardiovascular Medicine of our hospital from July 2021 to June 2023 were consecutively enrolled. The study protocol received approval from the Institutional Review Board. The diagnosis of severe coronary artery calcification lesions mainly relies on imaging methods, and commonly used methods include coronary artery computed tomography (CTA) examination, coronary angiography, and intravenous ultrasound (IVUS). The indications for RA mainly include: (1) severe calcification, that is, clear coronary artery calcification shadows can be seen before injection of contrast media; (2) when coronary angiography cannot determine whether the lesioned blood vessel is severely calcified, IVUS is used to examine the degree of coronary artery calcification. IVUS shows that strong echogenic light groups with acoustic shadows are distributed along the blood vessel wall. Lesions with an arc of calcification >270° are severely calcified; (3) balloon-noncompliant calcified lesions. Exclusion criteria: ① severe infection, severe liver and kidney dysfunction, severe heart failure, combined tumors and rheumatic autoimmune diseases; ② allergy to iodinated contrast media, use of iodinated contrast media or other contrast media in the past 2 weeks; ③ therapeutic use of nephrotoxic drugs such as aminoglycosides and nonsteroidal antiinflammatory drugs (NSAIDs) in the past week and within 72 hours after surgery; ④ oral metformin within 48 hours.

### 2.2 Clinical and Angiographic Data

The relevant data of the patients were collected. According to the CIN criteria developed by the European Society of Urogenital Radiology, the patients after PCI were stratified into the CIN and non-CIN groups. The risk factors for CIN after PCI in patients with coronary heart disease were analyzed. The basic information of the patients, including gender, age, hypertension, hyperlipidemia, diabetes, and smoking, was recorded. Venous blood was collected before admission to the hospital to test the levels of liver and kidney function, glomerular filtration rate, blood lipids, blood sugar, brain natriuretic peptide, and other indicators. Renal function was monitored within 48–72 hours after RA, and the highest creatinine value was recorded. All patients were treated with normal saline hydration at a rate of 1 mL/(kg⋅h) 12–24 hours after surgery.

### 2.3 Revascularization Methods and Processes

RA was performed via the femoral, radial, or brachial artery, with unfractionated heparin 70–100 U/kg given before surgery. The Boston Scientific Rotablator™ was used for RA, and the RA head was Rota Link™ (catalog number: H749236310030; diameters of 1.25 mm, 1.50 mm, and 1.75 mm). The size of the RA head was selected to have a ratio of 0.5–0.6 between the RA head diameter and the vessel diameter, and the RA speed was 140,000–160,000 rpm. Each RA procedure required 10–15 seconds. Throughout RA, a continuous intracoronary infusion combining unfractionated heparin and nitroglycerin was administered. Procedural success was defined by achieving complete balloon dilatation of the target lesion following RA. Before RA, all patients received 300 mg of aspirin and an oral loading dose of a P2Y_12_ receptor inhibitor (P2Y_12_, clopidogrel or ticagrelor). At discharge, all patients were maintained on a regimen of aspirin (100 mg daily) combined with either clopidogrel (75 mg daily) or ticagrelor (90 mg twice daily) for a minimum duration of 12 months.

### 2.4 Observation Indicators and Related Definitions

**Fig. 1.**
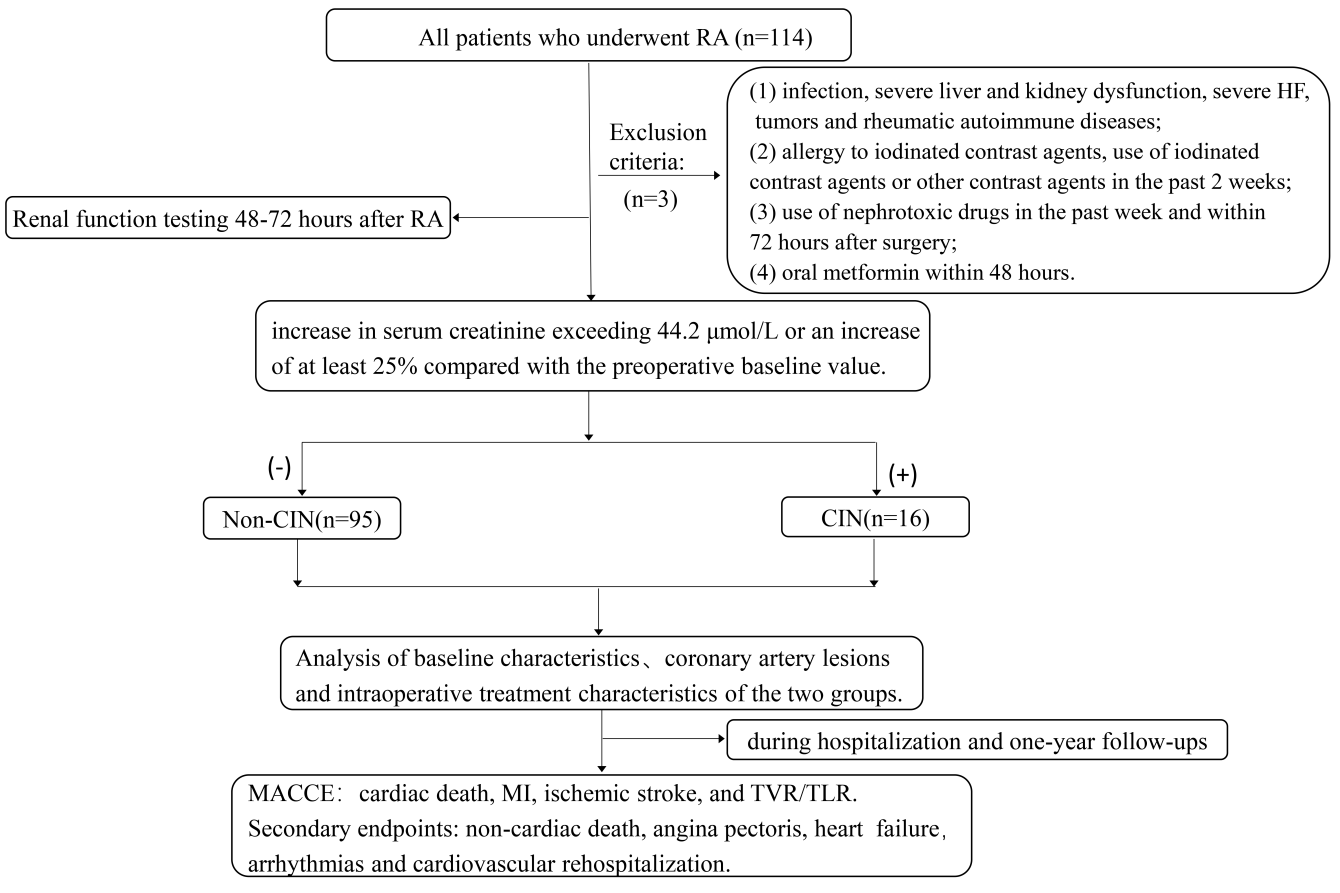
**Study flowchart**. RA, rotational atherectomy; HF, heart failure; CIN, contrast-induced nephropathy; MACCE, major adverse cardiovascular and cerebrovascular events; MI, myocardial infarction; TVR, target vessel revascularization; TLR, target lesion revascularization.

Acute renal impairment is detected within 48 to 72 hours after intravenous contrast media injection, as evidenced by an increase in serum creatinine exceeding 44.2 µmol/L or an increase of at least 25% compared with the preoperative baseline value. CIN can be diagnosed [12]. Perioperative myocardial infarction (MI) was defined as TnI exceeding 5 times the upper reference limit within 48 hours and new electrocardiogram (ECG) changes or imaging evidence after PCI [13]. According to the above criteria, patients were stratified into a CIN group (16 cases) and a non-CIN group (95 cases) (Fig. 1).

### 2.5 Adjust for Confounders

Given that the sample size of the non-CIN group is larger than compared of the CIN group, and greater inherent variability within the non-CIN group, propensity score matching (PSM) was implemented to enhance inter-group balance and statistical efficiency. Using propensity scores derived from gender, age, diabetes mellitus status, and preoperative glomerular filtration rate (GFR) as matching covariates with a caliper width of 0.02, 1:1 propensity score matching was performed. Each patient in the CIN group was thereby matched to a counterpart in the non-CIN group with identical or highly similar baseline characteristics, effectively minimizing confounding from extraneous factors.

### 2.6 Clinical Follow-up

Patients were tracked clinically at 1, 3, 6, and 12 months post-procedure. Clinical events were ascertained during follow-up, which was conducted via telephone interviews, clinic visits, or medical record review. All patients completed the follow-up. The composite primary endpoint—major adverse cardiovascular and cerebrovascular events (MACCE)—included cardiac death, spontaneous MI, ischemic stroke, or repeat target vessel revascularization/target lesion revascularization (TVR/TLR). Secondary endpoints comprised the composite of non-cardiac death, angina pectoris, heart failure, arrhythmias, and cardiovascular rehospitalization. All endpoints were adjudicated according to the standardized criteria proposed by the Cardiovascular Trials Initiative [14]. Deaths were categorized as cardiac or non-cardiac, with deaths of unknown etiology classified as cardiac deaths. Cardiac death was designated as mortality from cardiovascular causes, specifically: acute myocardial infarction, sudden cardiac death, heart failure, stroke, cardiovascular procedure-related complications, major cardiovascular hemorrhage, or other established cardiovascular origins. The clinical definition of MI denotes acute myocardial injury characterized by abnormal cardiac biomarkers occurring in the presence of evidence supporting acute myocardial ischemia. Stroke was defined based on the presence of acute infarction demonstrated by imaging or on the persistence of neurological symptoms consistent with stroke. TVR constituted repeat revascularization of the target vessel, whether performed by PCI or surgically. TLR was defined as any repeat revascularization, specifically of the target lesion, performed for restenosis or other complications related to that lesion, with the target lesion encompassing the treated segment extending 5 mm proximal and distal to the stent edges.

### 2.7 Statistical Analysis

Data analysis utilized SPSS 26.0 (IBM Corp., Armonk, NY, USA). Continuous variables following normal distributions were presented as mean ± SD and compared using independent *t*-tests. Non-normally distributed variables were expressed as median interquartile range (IQR) with Mann-Whitney U or Kolmogorov-Smirnov tests for group comparisons. Categorical data were reported as percentages (%) and analyzed via χ^2^ or Fisher’s exact tests. Significance was determined at two-tailed *p* < 0.05. Multivariate logistic regression evaluated CIN risk factors. PSM enables control of confounding factors, reduces potential bias affecting study outcomes between groups, and enhances the validity of results.

## 3. Research Results

### 3.1 Comparison of Baseline Data Between the Two Groups of Patients

The overall study population consisted predominantly of high-risk cardiovascular patients with at least two cardiovascular risk factors. All patients were diagnosed with acute coronary syndrome (ACS). The CIN group exhibited more pronounced cardiac and renal risk features compared to the non-CIN group. There were no significant differences in gender, age, diabetes, hypertension, history of stroke, history of PCI, preoperative MAP, and preoperative GFR across groups (*p* > 0.05). Patients in the CIN group exhibited significantly higher rates of both heart failure (84.6% vs. 38.5%; *p* < 0.05) and preoperative MAP <80 mmHg (53.8% vs. 7.7%; *p* < 0.05) compared to the non-CIN group. Prior to PSM, the non-CIN group demonstrated notably higher rates of unstable angina and lower incidence of non-ST-segment elevation myocardial infarction (NSTEMI) compared to the CIN group (*p* < 0.05). However, after PSM implementation, no significant differences emerged in either unstable angina or NSTEMI occurrence between the matched cohorts (*p* > 0.05, Table 1).

**Table 1.** **Baseline characteristics of patients in the two groups**.

Variables	Pre-PSM (n = 111)			Post-PSM (n = 26)		
	Non-CIN (n = 95)	CIN (n = 16)	*p* value	Non-CIN (n = 13)	CIN (n = 13)	*p* value
Age	68.4 ± 8.7	66.6 ± 14.5	0.46​	68.2 ± 9.3	71.9 ± 9.4	0.32​
Male	56 (58.9)	7 (43.8)	0.26​	7 (53.8)	5 (38.5)	0.70
Previous PCI	18 (18.9)	1 (6.3)	0.37​	4 (30.8)	1 (7.7)	0.32​
Smoking, n (%)	23 (24.2)	4 (25)	0.95​	3 (23.1)	3 (23.1)	1.00
Hypertension	83 (87.4)	15 (93.8)	0.75​	12 (92.3)	12 (92.3)	1.00​
Diabetes	40 (42.1)	12 (75)	0.03​	9 (69.2)	9 (69.2)	1.00​
ACEI	31 (32.6)	6 (37.5)	0.70	4 (30.8)	4 (30.8)	1.00
History of stroke	27 (28.4)	6 (37.5)	0.46​	5 (38.5)	5 (38.5)	1.00
Preoperative MAP (mmHg)	90.1 ± 11.6	83.8 ± 18.0	0.19	89.8 ± 8.9	86.2 ± 21.1	0.58
Preoperative MAP <80 mmHg	14 (14.7)	10 (62.5)	<0.001	1 (7.7)	7 (53.8)	0.03
Unstable angina	81 (85.3)	7 (43.8)	<0.001	11 (84.6)	7 (53.8)	0.20
Non-STEMI	14 (14.7)	9 (56.3)	<0.001	2 (15.4)	6 (46.2)	0.20
GFR	79.42 ± 27.7	61.60 ± 40.1	0.10​	73.0 ± 26.7	73.5 ± 34.5	0.97
Heart failure	30 (31.6)	14 (87.5)	<0.001	5 (38.5)	11 (84.6)	0.04

PSM, propensity score matching; CIN, contrast-induced nephropathy; PCI, percutaneous coronary intervention; ACEI, angiotensin-converting enzyme inhibitor; MAP, mean arterial pressure; Non-STEMI, non-ST-segment elevation myocardial infarction; GFR, glomerular filtration rate.

### 3.2 Analysis of Coronary Artery Lesions and Intraoperative Treatment Characteristics of the Two Groups of Patients

Prior to PSM: No statistically significant differences were observed between the groups in multivessel lesion rates, IVUS utilization frequency, number of pre-dilation balloons or stents deployed, procedure duration, or contrast media volume administered (all *p* > 0.05). Intraaortic balloon pump (IABP) use prevalence was significantly greater in CIN versus non-CIN cohorts (56.3% vs. 22.1%, *p* = 0.004), and rescue RA was utilized significantly more frequently in the CIN group compared to the non-CIN group (50.0% vs. 20.0%, *p* = 0.01).

After PSM: No statistically significant differences were observed in coronary lesion characteristics or intraprocedural interventions between the two patient groups (Table 2).

**Table 2.** **Analysis of the characteristics of coronary artery lesions and interventional treatment in the two groups of patients**.

Variables	Pre-PSM (n = 111)			Post-PSM (n = 26)		
	Non-CIN (n = 95)	CIN (n = 16)	*p* value	Non-CIN (n = 13)	CIN (n = 13)	*p* value
Multivessel	76 (80)	13 (81.3)	0.91​	12 (92.3)	10 (76.9)	0.59
IABP, n (%)	21 (22.1)	9 (56.3)	0.01	4 (30.8)	7 (53.8)	0.43
Rescue RA	19 (20)	8 (50)	0.01​	7 (53.8)	6 (46.2)	1.00
Intraoperative use of IVUS	16 (16.8)	3 (18.8)	1.00	2 (15.4)	3 (23.1)	1.00
Operation time	80.3 ± 25.8	86.9 ± 24.4	0.34	83.9 ± 25.8	89.2 ± 26.6	0.61
contrast media dose	174.4 ± 54.9​​	192.5 ± 46.1​​	0.22	200.8 ± 68.0​​	193.9 ± 46.3​​	0.77
Number of pre-dilation balloons	2.07 ± 1.28	2.38 ± 1.03	0.38	2.1 ± 0.9	2.5 ± 1.1	0.32
Number of brackets	1.65 ± 0.7	1.81 ± 1.0	0.42	1.6 ± 0.8	1.8 ± 0.8	0.63

PSM, propensity score matching; CIN, contrast-induced nephropathy; IABP, intraaortic balloon pump; RA, rotational atherectomy; IVUS, intravascular ultrasound.

### 3.3 Analysis of Influencing Factors of CIN in Patients Undergoing RA

CIN after RA was taken as the dependent variable, and the indicators with differences between the two groups, including heart failure, preoperative MAP, and preoperative diagnosis, were taken as independent variables. After PSM, the results of logistic regression showed that preoperative MAP <80 mmHg (OR = 17.865, 95% CI: 1.135–281.246) was an influencing factor for the incidence of CIN after RA (*p* < 0.05), as shown in Table 3.

**Table 3.** **Multifactorial logistic regression model of CIN risk**.

	Influencing factors	β	SE	Wald χ^2^	OR (95% CI)	*p*
Pre-PSM	Heart failure	1.861	0.943	3.896	6.430 (1.013–40.809)	0.048
	MAP <80 mmHg	1.658	0.761	4.751	5.250 (1.182–23.318)	0.03
	Non-STEMI	0.521	0.845	0.380	1.683 (0.321–8.825)	0.54
Post-PSM	Heart failure	2.377	1.330	3.195	10.778 (0.795–146.122)	0.07
	MAP <80 mmHg	2.883	1.406	4.202	17.865 (1.135–281.246)	0.04
	Non-STEMI	0.113	1.226	0.008	1.119 (0.101–12.375)	0.93

PSM, propensity score matching; MAP, mean arterial pressure; Non-STEMI, non-ST-segment elevation myocardial infarction; SE, standard error; OR, odds ratio; CI, confidence interval.

### 3.4 Clinical Events in Both Groups of Patients During Hospitalization and One Year After RA

Prior to PSM: The incidence of periprocedural MI was significantly higher in the CIN group compared with the non-CIN group (*p* < 0.05). All patients completed the 1-year follow-up, and no data were missing during the study period. MACCE occurred in five patients in the non-CIN group, whereas no MACCE events occurred in the CIN group (*p* = 0.77). A significant difference was observed in cardiovascular rehospitalization rates between the two groups (8.4% vs. 31.3%, *p* = 0.01), while no statistically significant differences were found for the other secondary endpoints (unstable angina, dialysis). Six non-cardiac deaths occurred in the non-CIN group and two in the CIN group (*p* = 0.72), all attributed to pulmonary infection.

After PSM: No statistically significant differences were observed in MACCE or the secondary endpoint between the two patient groups (Table 4).

**Table 4.** **Clinical events in both groups during hospitalization and one year after RA**.

Pre-PSM			
Variables	Non-CIN (n = 95)	CIN (n = 16)	*p*
Periprocedural MI	6 (6.3)	4 (26.7)	0.04
MACCE	5 (5.3)	0 (0.0)	0.77
Unstable angina	2 (12.5)	17 (17.9)	0.86
Dialysis	0 (0.0)	1 (6.3)	0.54
Cardiovascular rehospitalization	8 (8.4)	5 (31.3)	0.01
Non-cardiac death	6 (6.3)	2 (12.5)	0.72
Post-PSM			
Variables	Non-CIN (n = 13)	CIN (n = 13)	*p*
MACCE	1 (7.7)	2 (15.4)	1.00
Secondary endpoint	6 (46.2)	4 (30.8)	0.69​

PSM, propensity score matching; CIN, contrast-induced nephropathy; MI, myocardial infarction; MACCE, major adverse cardiovascular and cerebrovascular events.

## 4. Discussion

Limited data exist on risk factors for CIN following RA. Our findings revealed that a history of diabetes, a history of hypertension, preoperative GFR, the presence or absence of heart failure, intraoperative use of IABP, and contrast volume were not risk factors for CIN in patients undergoing RA procedures. However, a preoperative MAP <80 mmHg was significantly associated with an increased risk of postoperative CIN. After adjusting for confounders, the incidence of MACCE and secondary endpoints in the CIN group was comparable to that in the non-CIN group. Consequently, patients with preoperative MAP <80 mmHg undergoing RA require intensified vigilance—including proactive screening and early intervention for CIN—to mitigate associated risks.

CIN and cholesterol crystal embolism (CCE) can both manifest as renal insufficiency following vascular interventional procedures, yet they differ fundamentally in nature. CIN is essentially an acute toxic injury to the renal tubules, induced by the direct cytotoxicity of iodinated contrast agents and ischemia in the renal medulla. It has a short latency period (24–72 hours), presenting as a transient elevation in serum creatinine (Scr) that typically resolves within 7–10 days in most patients. Urinary sediment may show renal tubular epithelial cells. It lacks extrarenal manifestations. The primary risk factors are pre-existing renal insufficiency and diabetes. CCE, conversely, is fundamentally an embolic vascular injury. It originates from the rupture of atherosclerotic plaques, leading to cholesterol crystals obstructing the renal arterioles. CCE usually becomes clinically evident after a relatively long latency period, typically presenting with a progressive and often irreversible increase in Scr within 1 to 4 weeks after contrast administration [15]. In this study, all 16 patients with CIN exhibited elevated Scr within 24 to 72 hours after surgery. One of these patients, who required permanent dialysis, already had stage 3b renal function before the procedure. None of the CIN patients developed peripheral eosinophilia or showed systemic embolic manifestations involving multiple organs, such as livedo reticularis (a net-like bluish-purple rash), digital gangrene, intestinal ischemia, or retinal Hollenhorst plaques—systemic features that may occur in CCE. Definitive exclusion of CCE may ultimately rely on biopsy (of skin, muscle, or kidney), where “needle-shaped clefts” left behind after the dissolution of cholesterol crystals can be observed within small arteries.

Literature shows that one-fifth of patients undergoing PCI have moderate or severe coronary artery calcification [11]. Using RA to treat severely calcified coronary lesions can make the stent obtain a larger diameter, a larger lumen cross-sectional area, and less final residual stenosis [10]. Patients undergoing RA also have a higher procedural success rate [3, 11]. Given these advantages, a growing number of interventional cardiologists now utilize RA for severely calcified coronary lesions. However, RA is related to higher technique demand, prolonged procedural time, higher radiation dose, and contrast media dose [3]. Clinical data are lacking regarding whether longer operation time and higher contrast media dose causally affect the incidence of CIN, the incidence, and related risk factors of CIN in patients undergoing RA. Our results showed that among 111 coronary heart disease patients who underwent RA, 16 developed CIN, representing an incidence rate of 14.41%. Notably, a previous study reported a CIN incidence rate of 19.51% [16] in coronary heart disease patients after PCI. Although the CIN incidence observed in this study is numerically similar to or a little bit lower compared to historical data, indicating similar incidence with or without RA, directly comparing the incidence of CIN is not possible because the characteristics of patients in this and previous studies might be different. The data should thus be interpreted with great caution due to potential significant differences in patient baseline characteristics.

MAP is an important physiological index reflecting organ perfusion, and it has a complex relationship with the occurrence and development of CIN. The pathological mechanism of CIN involves renal hemodynamic disturbances, tubular toxicity damage, and oxidative stress [12, 13, 17, 18], the risk of CIN through multiple pathways. A critically low MAP may significantly reduce renal blood flow, compromising the kidney’s autoregulatory capacity—particularly the balance of oxygen supply and demand in the renal medulla [10]. Studies indicate that contrast media can cause transient early vasodilation followed by sustained vasoconstriction, further diminishing medullary blood flow [19, 20]. Under low MAP conditions, this process exacerbates medullary hypoxia and increases the risk of acute kidney injury [21]. Our study also confirmed that the incidence of CIN in patients with concomitant heart failure (84.6%) was significantly higher than that in the non-heart failure group (38.5%). Early studies indicate that hemodynamic alterations secondary to cardiac dysfunction—specifically reduced cardiac output and intravascular volume—represent a key underlying mechanism of CIN [22, 23]. The hemodynamic alterations stemming from cardiac dysfunction—primarily characterized by diminished cardiac output and reduced intravascular volume—establish a direct pathophysiological link to decreased MAP. According to the fundamental equation MAP = Cardiac Output (CO) × Systemic Vascular Resistance (SVR), reductions in CO directly lower MAP. And MAP values <80 mmHg in such patients correlate with heightened risks of renal hypoperfusion and contrast-induced nephropathy.

Optimized hemodynamic management serves not only renal protection but also as a core cardiorenal preservation strategy. Given the critical importance of maintaining optimal MAP, continuous hemodynamic monitoring and targeted titration of MAP are clinically imperative, as both hypotension and hypertension can compromise renal function through distinct mechanisms. Comprehensive risk mitigation thus necessitates holistic evaluation of comorbidities, hemodynamic profiles, and contrast exposure parameters, integrating MAP optimization, adequate hydration, and personalized pharmacotherapy. To operationalize this paradigm, patients undergoing RA procedures require preoperative risk stratification, perioperative titration of MAP, and postoperative renal function monitoring. Furthermore, large-scale multi-center studies are needed to establish population-specific MAP targets and evidence-based intervention thresholds [24]. Ultimately, modulation of this modifiable risk factor promises significant reductions in CIN and associated cardiorenal adverse events.

## 5. Study Limitations

Shortcomings and prospects of the present study: (1) The single-center design and limited cohort constrain the generalizability of these observations. External validation through multi-center collaborations is imperative. (2) At present, the pathological and physiological mechanisms of CIN after PCI have not been fully elucidated, and further research is still needed in subsequent studies.

## 6. Conclusion

Once CIN is established, only supportive care is currently provided until renal function resolves. Therefore, presently, the main method to tackle this complication is its prevention. Preprocedural MAP <80 mmHg constitutes an independent risk factor for CIN following coronary RA, establishing a pivotal therapeutic target for precision perioperative management. This evidence mandates the integration of preoperative blood pressure assessment into standardized RA patient risk stratification protocols. Early identification of high-risk cohorts (MAP <80 mmHg) enables proactive intervention during the preprocedural window through individualized volume expansion and judicious vasoactive agent administration to elevate MAP to the safety threshold (≥80 mmHg), thereby intercepting CIN pathogenesis at its origin. Therefore, for patients undergoing coronary RA with preoperative MAP <80 mmHg, intensified perioperative MAP monitoring and early implementation of preventive measures against CIN are essential to mitigate the risk of adverse clinical events.

## Availability of Data and Materials

The datasets used and analyzed during the current study are available from the corresponding author on reasonable request.
